# The Sufferings of Christ

**Published:** 1888-04-21

**Authors:** 


### Words of Consolation.

### LXXIV.—The Sufferings of Christ.

*For Reading to the Sick.*

Following out the reasons for our Lord's dissolution, I will now call your attention to an old commandment, and then to a new one. The new one you probably know about, whether you have obeyed it or not; but its meaning cannot be fully grasped without some attention to the other, which is as follows: "Whatsoever man there be of the house of Israel, or of the strangers that sojourn among you, that eateth any manner of blood; I will even set my face against that soul that eateth blood, and will cut him off from among his people. For the life of the flesh is in the blood: and I have given it to you upon the altar to make an atonement for your souls. . . . No soul of you shall eat blood . . . for the life of all flesh is the blood thereof" (Lev. xvii. 10—14). This is but an expansion of the commandment given to Noah: "But flesh with the life thereof, which is the blood thereof, shall ye not eat" (Gen. ix. 4). Why were the flesh and blood not to be eaten? Because they represented the life; and the life was due to God. All the old sacrifices showed that life was forfeited. It no longer belonged to man. He had forfeited his right to it when he sinned. If therefore "the life" ever came back to him, it must come in the shape of a gift, and that gift must spring out of the Great Atonement. The man is first of all cut off from the tree of life. Then he is taught that a sacrifice must be offered for sin. But he learns at the same time that he has no right whatever to the life which flows from the sacrifice. A most awfully impressive object lesson was thus conveyed at the steps of the altar. The blood is forbidden. Now you may better understand the new commandment: "Come (saith Jesus), eat of my flesh and drink of my blood. Behold I have purchased the forfeited life for you. I have poured it forth not as waste: for it is my own, yet given for you. Come, partake of the flesh and blood which I said I would give for the life of the world. The blood is the life shed for many for the remission of sins. Drink ye all of it. My flesh is meat indeed, and my blood is drink indeed."

You are not to think here of the drops of blood which fell from the cross more than 1,800 years ago. No. Rather have you to think of the essence of life which has come forth to us from Christ's dissolution. This is the life which, after a heavenly and spiritual manner, is transmitted to the faithful in the Sacrament of the Lord's Supper. Let us understand that what took place strictly on the cross provided a full, perfect, and sufficient sacrifice for sin. But when we talk about faith in the blood of Christ now, that is, in its present efficacy, let us be clear. This should mean that we believe in His having poured forth His life with a view to our actual participation in it, just as we actually participate in the fruits of the earth which spring from the death of the corn seed. One feeding is natural, the other is spiritual. But do not suppose that the spiritual food is any the less real because it is given after such a heavenly and mysterious manner. Feeding, both in the natural and in the spiritual kingdom, is essential to the preservation of life. Starvation and death follow upon neglect in either case. We have no reason for supposing that spiritual life is exempt from the reign of law. The wasting and consequent starvation of multitudes of professing Christians, through disobedience to this law, is very grievous to think of. "Ye will not come unto me that ye might have life" (John v. 40).

